# Identification of a predictive gene signature related to pyroptosis for the prognosis of cutaneous melanoma

**DOI:** 10.1097/MD.0000000000030564

**Published:** 2022-09-09

**Authors:** Zhaoyang Shi, Jiaying Gu, Yi Yao, Zhengyuan Wu

**Affiliations:** a Department of Hand Plastic Surgery, The First People’s Hospital of Linping District, Hangzhou, China; b Department of Laboratory, Integrated Traditional Chinese and Western Medicine Hospital of Linping District, Hangzhou, China.

**Keywords:** bioinformatic analysis, cutaneous melanoma, prognosis, pyroptosis

## Abstract

Pyroptosis is a form of inflammatory programmed cell death. However, because of no specific molecular biomarker, pyroptosis has not been considered as a novel therapeutic method to treat cutaneous melanoma (CM). Here, we identified pyroptosis genes that associate with the prognosis of CM patients and constructed an effective model for the prognostic prediction of CM patients. To identify genes related to pyroptosis that are differentially expressed in CM, we obtained gene expression data of CM patients and normal skin tissues from the Cancer Genome Atlas and the Genotype-Tissue Expression databases, and used another cohort obtained from Gene Expression Omnibus database for validation. Three genes (*BST2*, *GBP5*, and *AIM2*) that were associated with prognosis were found and incorporated into our prognostic model. Furthermore, we divided the patients into 2 groups: a high-risk group and a low-risk group. Functional analyses indicated that our model was correlated with patient survival and cancer growth. Multivariate and univariate Cox regressions revealed that the constructed model could serve as an independent prognostic factor for CM patients. Meanwhile, compared with other clinical characteristics, our model significantly improved the diagnostic accuracy. Gene function analysis revealed that pyroptosis genes *BST2, GBP5*, and *AIM2* were differentially expressed in CM patients and positively associated with patient prognosis. Finally, a risk score was used to generate nomograms that displayed favorable discriminatory abilities for CM. In summary, our model could significantly predict the prognosis of CM patients and be used for the development of CM therapy.

## 1. Introduction

Cutaneous melanoma (CM) is an aggressive malignant tumor that threatens human life.^[1]^ Based on existing investigations, 287,723 people were diagnosed with melanoma worldwide, and 60,709 died due to this disease in 2018.^[2]^ Recently, numerous experimental and clinical studies have demonstrated novel drugs for cancer treatment, such as extracts and cucurbitacin B from Luffa operculata (L.) Cogn,^[3]^ essential oils from Ipomoea L. species,^[4]^ chalcones,^[5]^ methoxylated fraction from *Vellozia dasypus* Seub,^[6]^ eleutherin and isoeleutherin from *Eleutherine plicata*,^[7]^ and so on. However, due to the pathogenic complexity of CM and the precise pathogenesis behind the disease is still unknown, there is currently no effective treatment. Meanwhile, according to the report, the 10-year overall survival (OS) rates of CM patients in stages I and II are respectively 75% to 98%.^[8,9]^ In contrast, compared with CM patients in stages I and II, only 24% to 88% of CM patients in stages IIIA to IIID survived after 10 years, suggesting that the early diagnosis of CM may affect its outcome. Many investigators have attempted to identify novel biomarkers that can be used for the prognostic prediction and personalized therapy of CM patients, however, only a few biomarkers of clinical significance were identified.^[10]^ Therefore, the identification of new biomarkers that can accurately predict the prognosis of CM patients is urgently needed.

Pyroptosis is a new type of inflammatory programmed cell death.^[11]^ Gasdermin D (GSDMD) is regarded as the primary effector of pyroptosis.^[12]^ In this process, inflammasomes are formed and activated by recruiting inflammatory caspases to multiprotein complexes. AIM2-like receptors and NOD-like receptors are cytosolic pattern recognition receptors and can trigger the assembly of multiprotein complexes.^[13]^ Meanwhile, AIM2-like receptors and NOD-like receptors are also key components of inflammasomes, accompanied by pro-Caspase-1 and apoptosis-associated speck-like protein.^[14]^ Inflammasomes stimulate the activation of cysteine protease Caspase-1, which can subsequently induce pro-IL-18 and pro-IL-1β maturation, and the release of GSDMD.^[15]^ Then, GSDMD is multimerized and sheared, forming several 10–20 nm pores in the cell membrane. Cell content can pass through these membrane pores, in which cells will produce apoptotic vesicle-like protrusions and gradually swell until rupture.^[16,17]^ Meanwhile, IL-18 and IL-1β can also be released from these pores. Pyroptosis pathways are classified as classical or nonclassical, depending on the recognition of damage signals by different cytoplasmic protein sensors. In recent years, a growing number of reports show that pyroptosis is significantly involved in the pathogenesis and progression of CM. It has been verified that components of pyroptosis, namely inflammatory vesicles, cytokines, and genes of the gasdermin family were significantly involved in tumorigenesis and metastasis of melanoma.^[18]^ In comparison to traditional cell apoptosis, increased activation of the immune system and inflammatory responses were induced by cells under pyroptosis, further mechanistic studies revealed that this was triggered by factors released by pyroptotic cells.^[19]^ Additionally, researchers also found that pyroptosis could exert its antitumor effect through the tumor immune microenvironment (NK cells^[20]^ and CD8 + T lymphocytes^[21]^). Thus, it is widely accepted that pyroptosis is relevant for tumor diagnosis and therapy, however, its specific role in CM is still uncovered.

Several disease-specific biomarkers were identified using bioinformatics tools; however, the use of a single analysis approach hinders the discriminatory capacity of highly connected genes. Additionally, intergenomic epistasis has been often ignored. Therefore, in this study, we identified the genes differentially expressed that associate with pyroptosis in CM patients using weighted gene co-expression network analysis (WGCNA). An analysis which identifies the differentially expressed genes was employed to enhance the highly connected genes’ discriminatory ability. Subsequently, least absolute shrinkage and selection operator (LASSO) and univariate Cox regression were performed to characterize hub genes significantly related to CM prognosis. Therefore, based on hub genes, a risk model using for prognositic prediction was constructed to explore its value in CM patients. Currently, most existing models are generated according to tumor immunity and miRNAs, while a thorough analysis of pyroptotic genes has not been performed yet. Thus, we present the value of pyroptosis-associated genes as predictive prognostic tools for CM patients.

## 2. Materials and Methods

### 2.1. Datasets

Clinical information and RNA-sequencing data of CM patients (n = 471) and normal control (n = 1) were obtained from the University of California Santa Cruz Xena on June 30, 2020 (UCSC Xena). Transcriptome data for 812 normal skin samples were downloaded from genotype-tissue expression. Meanwhile, clinical information and gene profiles of CM patients (n = 214) were obtained from the Gene Expression Omnibus database (ID: GSE65904) and were used as a cohort for external validation. In addition, to remove batch effects, log2-transformation and normalization were conducted using the “sva” package. Furthermore, the GeneCards database was used to collect protein domains of pyroptosis genes (n = 146). This study was reviewed and approved by the Medical Ethics Committee of the First People’s Hospital of Linping District (ethic cord: 2021 (KY-E-018)). All experiments were performed in accordance with relevant guidelines and regulations. Informed consent was obtained from all subjects and/or their legal guardian(s) in case of participants <18 years of old were involved.

### 2.2. Construction of WGCN and identification of the hub gene module

WGCN was employed to analyze the impact of genes on phenotypic traits.^[22]^ Therefore, a gene co-expression networking was generated through the “WGCNA” package using pyroptosis genes in The Cancer Genome Atlas (TCGA)-CM cohort. Genes were tested for pairwise Pearson correlation. The following formula was used to identify a weighted adjacency matrix: amn=|cmn|β (cmn = Pearson correlation between gene n and gene m; amn = adjacency between gene m and gene n). Next, “β” was determined and used to identify strong gene correlations. A topological overlap matrix transformed the adjacencies. Average linkage hierarchical clustering was used to construct dendrograms with at least 10 modules. Dissimilarities of module eigengenes were calculated. Furthermore, modules relevant to CM clinical traits were identified through their eigengenes and gene significance. Genes in the functionally relevant module were further analyzed.

### 2.3. Analysis of expression and interactions

Differentially expressed pyroptosis genes (DEPGs) were identified by a differential expression analysis using the “limma” package. Candidate DEPGs were defined as the genes with a |log2 fold change| > 1 and false discovery rate < 0.05 based on a previous report and were visualized in heatmap and volcano plots using the “pheatmap” and “ggplot2” packages, respectively. Furthermore, genes overlapping between the WGCN, candidate DEPGs, and GSE65904 were considered “real” DEPGs and were visualized in a Venn diagram using the “VennDiagram” package.^[23]^

### 2.4. Construction and evaluation of the prognostic model

Univariate Cox regressions were performed for all DEPGs using the “survival” package with a cutoff of *P* < .05. After that, pyroptosis genes were integrated into a LASSO analysis to identify hub pyroptosis genes and generate a CM risk signature. Next, CM patients were divided into 2 groups based on their risk scores. The patient risk score was calculated as:


$$\begin{array}{l} \text{risk }\;\;\;\;\;\text{ score}=\;\;\;\Sigma \;\;\text{ expgenei* }\;\;\beta \;\;\text{ i} \end{array}$$

where expgenei represents the relative expression of pyroptosis genes, i and β are regression coefficients. Next, the “survival” package was applied to compare outcomes according to risk levels. To calculate the predictive accuracy of the model, we used the “survival receiver operating characteristic” and “time receiver operating characteristic” packages. Finally, Cox regression analyses were employed to evaluate the prognostic ability of the model, while a nomogram incorporating calibration plots was constructed using the “rms” package to predict patient outcome.

### 2.5. Evaluation of hub genes

Differences in protein expression of hub pyroptosis genes were determined by the Human Protein Atlas online database (http://www.proteinatlas.org/). The “ggpubr” package was applied to confirm the expression of hub pyroptosis genes from the TCGA-CM cohort. The prognostic value of hub genes was determined by the Kaplan-Meier method using the TCGA-CM cohort.

### 2.6. Enrichment analyses of GO and KEGG

The Integrated Discovery, Visualization, and Annotation database (version 6.8) was employed using gene ontology and Kyoto Encyclopedia of Genes and Genomes enrichment analyses in order to establish the biological function of pyroptosis genes. Statistical significance was reached when both false discovery rate and *P* were <.05.

## 3. Results

### 3.1. New pyroptosis gene hub modules using WGCN

**Figure 1.**
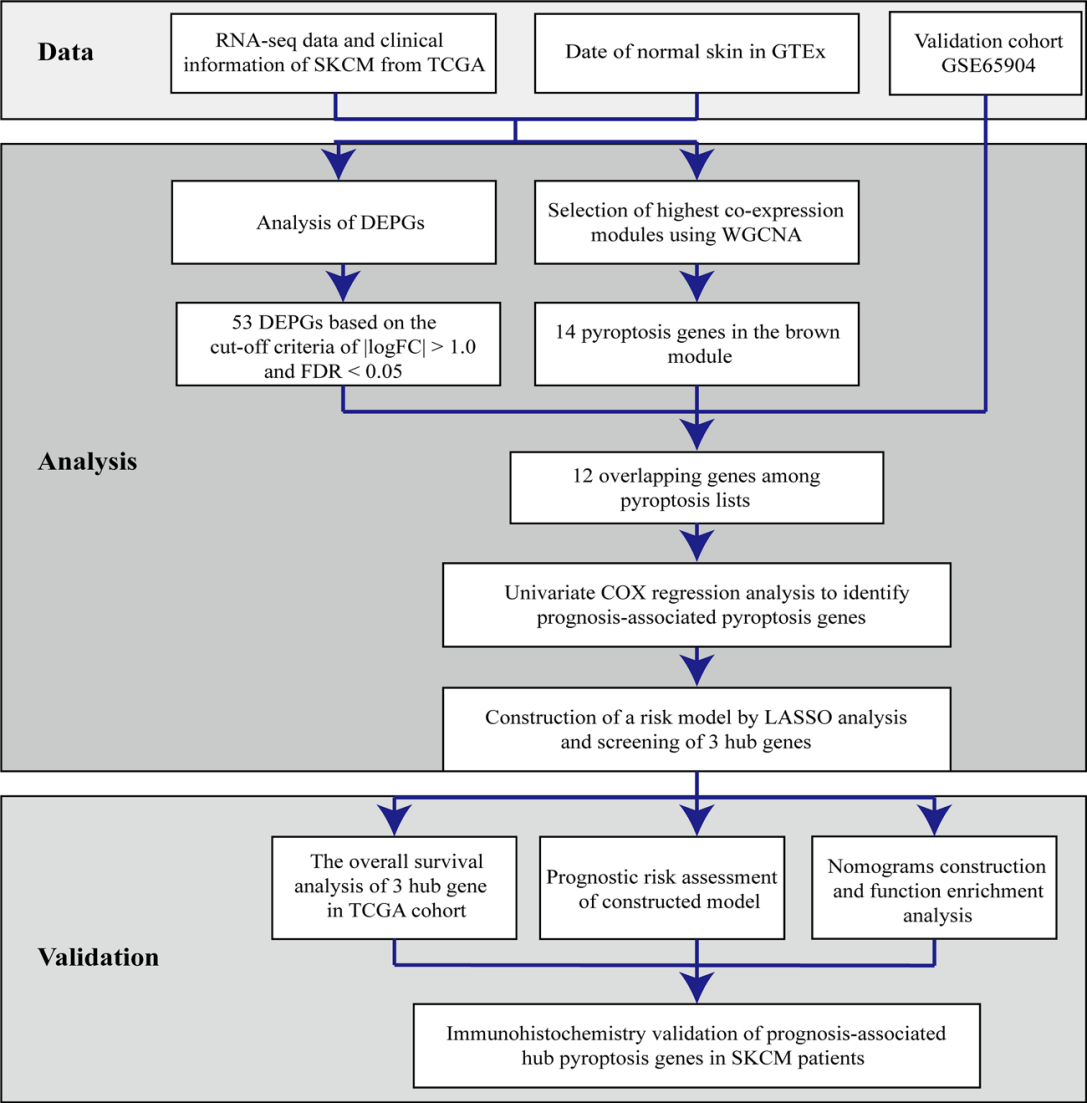
Schematic design of the study.

**Figure 2.**
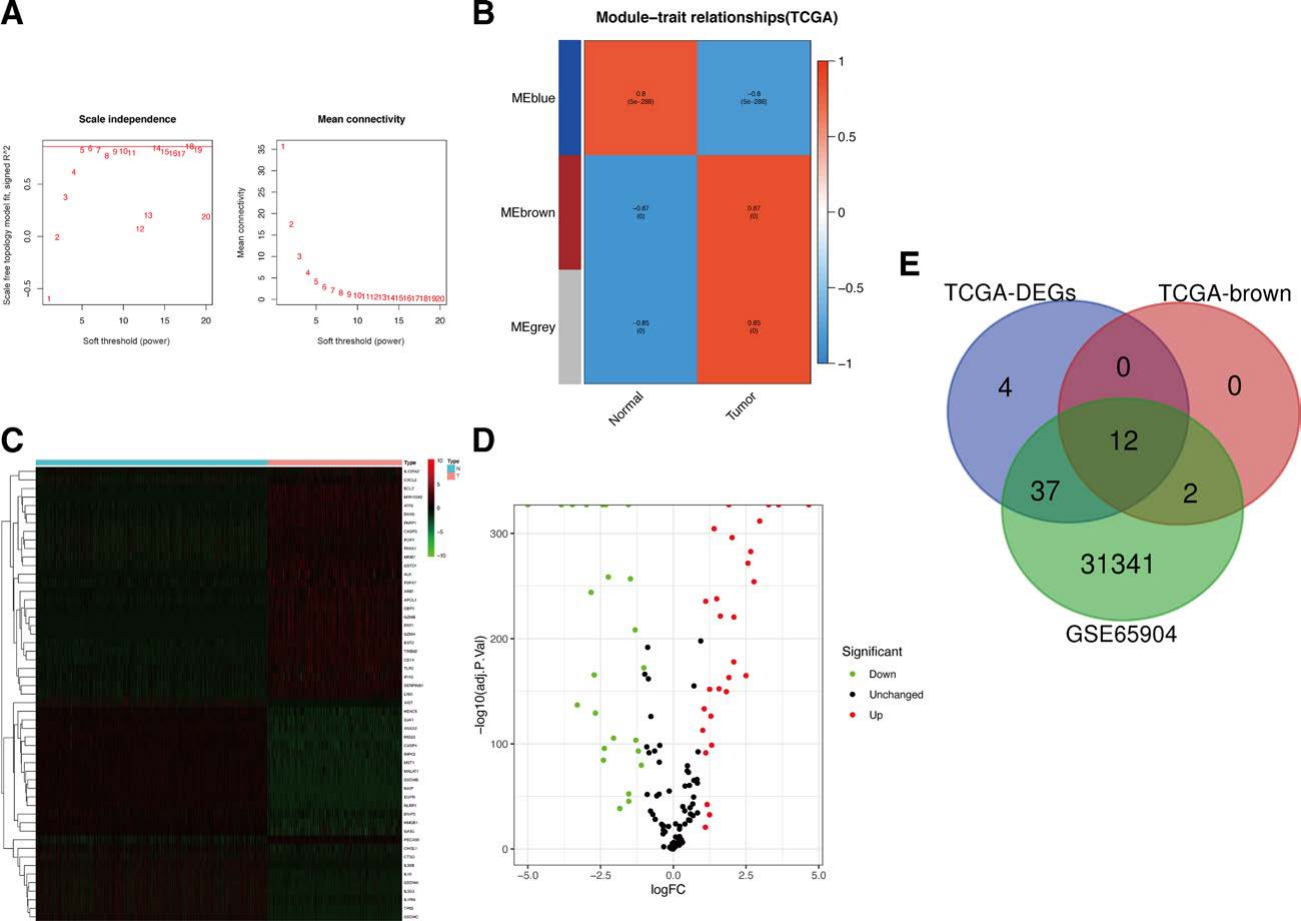
Characterization of DEPGs among the TCGA and GSE65904 cohorts of CM. (A) Soft-thresholding powers scale-free fit index. (B) Heatmap showing the correlation between clinical traits and gene module. Each module was assigned with different colors. The correlation coefficient decreased in size from red to blue. Heatmap (C) and volcano plot (D) of candidate DEPGs in the TCGA dataset with FDR < 0.05 and |logFC| > 1. (E) The Venn diagram of genes among candidate DEPG, WGCN, and GSE65904 lists. CM = cutaneous melanoma, DEPGs = differentially expressed pyroptosis genes, FDR = false discovery rate, TCGA = The Cancer Genome Atlas.

Figure 1 presents all datasets analyzed. In order to identify functional clusters, 471 CM samples from the TCGA-CM cohort were used for the WGCNA analysis of 146 extracted pyroptosis genes. The soft threshold was defined as β = 18 (scale-free R^2^ = 0.86) for the scale-free network (Fig. 2A). Three identified co-expressed modules were assigned with different colors to visualize connections with CM or normal traits. The brown module showed the closest association with tumor or normal tissues (r = −0.87, *P* = 0; Fig. 2B). Thus, this module was selected for further analysis.

### 3.2. Overlapping DEPGs

Based on the analysis of DEPGs, 53 pyroptosis genes were identified within the TCGA dataset. Among these, 25 pyroptosis genes were downregulated and 28 were upregulated (Fig. 2C,D). The distribution of coexpression genes from the GSE65904 cohort, the brown module from the TCGA-CM cohort, and DEPGs candidates are shown in Figure 2E. Altogether, a total of 12 overlapping pyroptosis genes were identified.

### 3.3. Screening for prognosis-associated pyroptosis genes and construction of a genetic score model for CM patients

**Figure 3.**
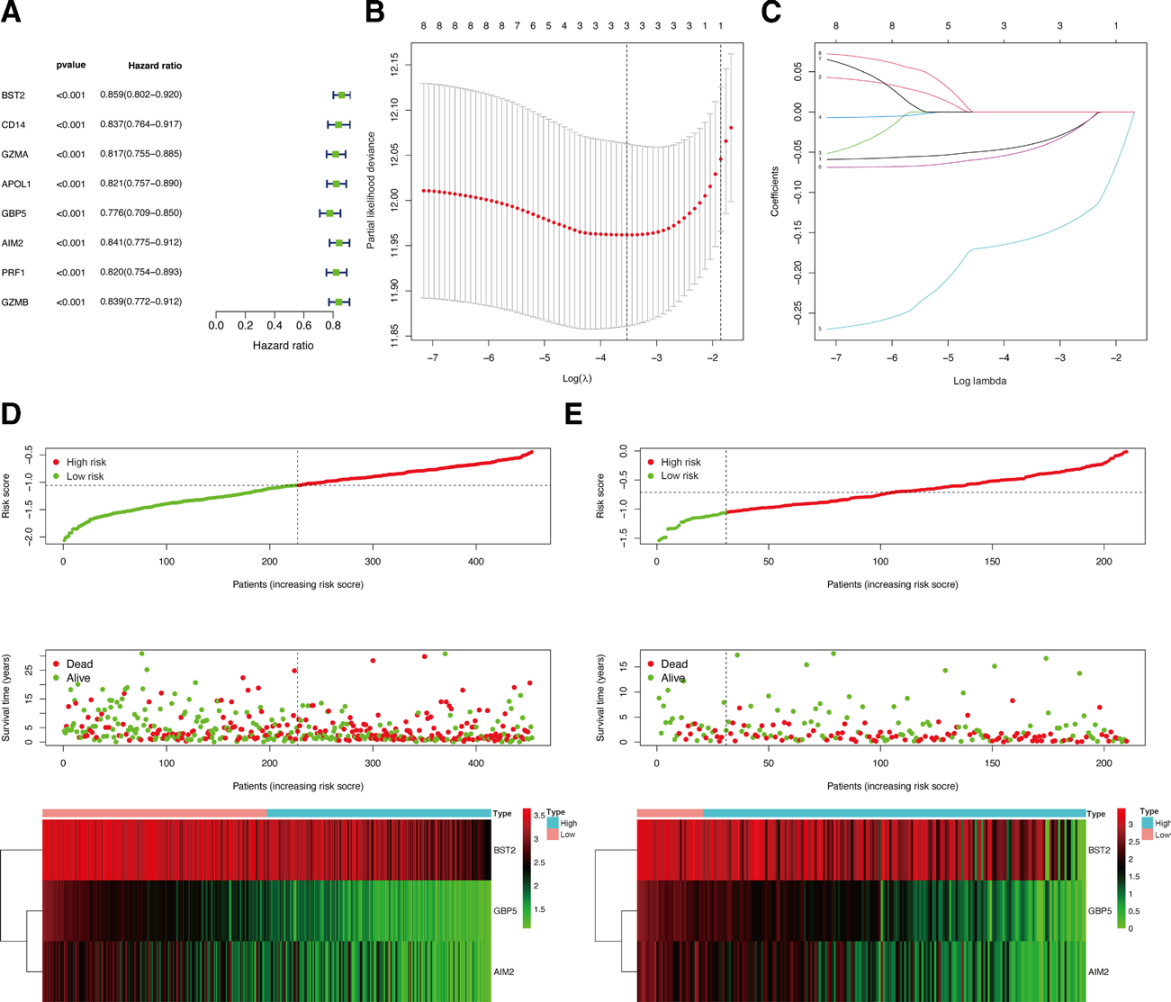
Construction of a predictive model using the TCGA and GSE65904 cohorts. (A) Univariate Cox regressions for prognosis-related pyroptosis genes in the TCGA cohort. (B, C) LASSO analysis to determine factors and construct the model. (D) Data from TCGA: survival, risk score, and expression heat map. (E) Data from TCGA: survival, risk score, and expression heat map. LASSO = least absolute shrinkage and selection operator, TCGA = The Cancer Genome Atlas.

The 12 identified DEPGs were further analyzed by univariate Cox regressions and 8 pyroptosis genes that showed *P* < .05 were selected (Fig. 3A). Then, the LASSO analysis was applied and 3 hub pyroptosis genes, namely *BST2, GBP5*, and *AIM2* were used to construct the risk signature model (Fig. 3B, C). Patients in TCGA-CM (Fig. 3D) and GSE65904 (Fig. 3E) cohorts were classified as low- or high-risk subgroups based on their median risk scores. The coefficients of pyroptosis genes used in the risk signature model are presented in Table 1.

**Table 1:** Three prognosis-associated pyroptosis genes in the TCGA-CM cohort were identified by LASSO analysis.

OS name	Univariate Cox regression analysis				LASSO coefficient
	HR	Lower 95% CI	Upper 95% CI	p-value	
BST2	0.8593	0.8022	0.9203	0.0000	-0.0452
GBP5	0.7761	0.7089	0.8497	0.0000	-0.1636
AIM2	0.8406	0.7747	0.7536	0.0000	-0.0531

CM = cutaneous melanoma, LASSO = least absolute shrinkage and selection operator, TCGA = The Cancer Genome Atlas.

### 3.4. Correlations between risk scores and clinical characteristics in CM patients

**Figure 4.**
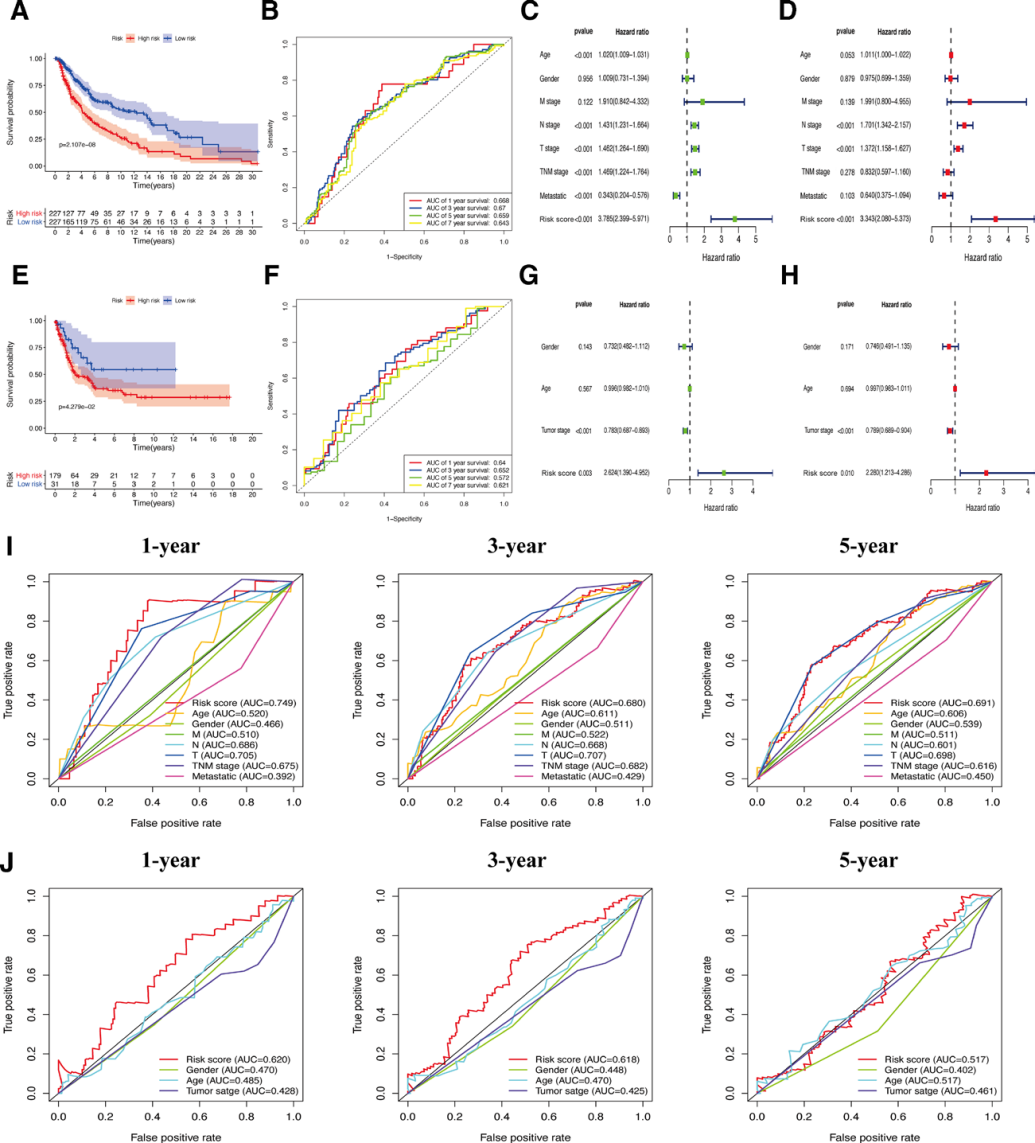
Efficacy of the constructed model. (A) Survival curve of the TCGA cohort. (B) TimeROC curves to forecast the overall survival (TCGA). (E) Survival curve of the GSE65904 cohort. (F) TimeROC curves to forecast the overall survival (GSE65904). Univariate and multivariate Cox regressions of clinicopathological features in TCGA (C, D) and GSE65904 (G–H). ClinicalROC curves to forecast the overall survival using data from TCGA (I) and GSE65904 (J). ROC = receiver operating characteristic, TCGA = The Cancer Genome Atlas.

The OS of high-risk CM patients was significantly lower than that of low-risk subgroup in the TCGA cohort (*P* < .001; Fig. 4A). Multivariate and univariate Cox regressions showed that, in CM patients, the risk score was an independent prognostic factor, whereas the risk signature was associated with prognosis (Fig. 4C, D). In the validation cohort, although the Kaplan-Meier analysis had a larger *P* value than the TCGA cohort, the OS of CM patients in high-risk subgroup was still significantly lower than that of low-risk subgroup (*P* < .05; Fig. 4E), this may be caused by the insufficient sample size of CM patients in the validation cohort. In Figure 4G and H, it was confirmed that the identified signature could be considered as an independent prognostic factor while compared with clinical features of gender, age, and tumor stages in the GSE65904 cohort (*P* < .05). Additionally, receiver operating characteristic analysis indicated that our risk signature contained a moderate predictive accuracy at 1-year (area under the ROC curve (AUC) = 0.668), 3-year (AUC = 0.670), 5-year (AUC = 0.659), and 7-year (AUC = 0.643) follow-up in the TCGA-CM cohort (Fig. 4B). Furthermore, compared with other clinical characteristics, our risk signature had a higher predictive power (Fig. 4I). The AUC was slightly decreased in the validation cohort, in which AUC = 0.64 for 1-year, AUC = 0.652 for 3-year, AUC = 0.572 for 5-year, and AUC = 0.621 for 7-year follow-up (Fig. 4F). However, its predictive accuracy was still higher than that of other clinical features at every follow-up time point. (Fig. 4J). This corroborates that our risk signature model could sensitively and specifically predicts the OS of CM patients.

**Figure 5.**
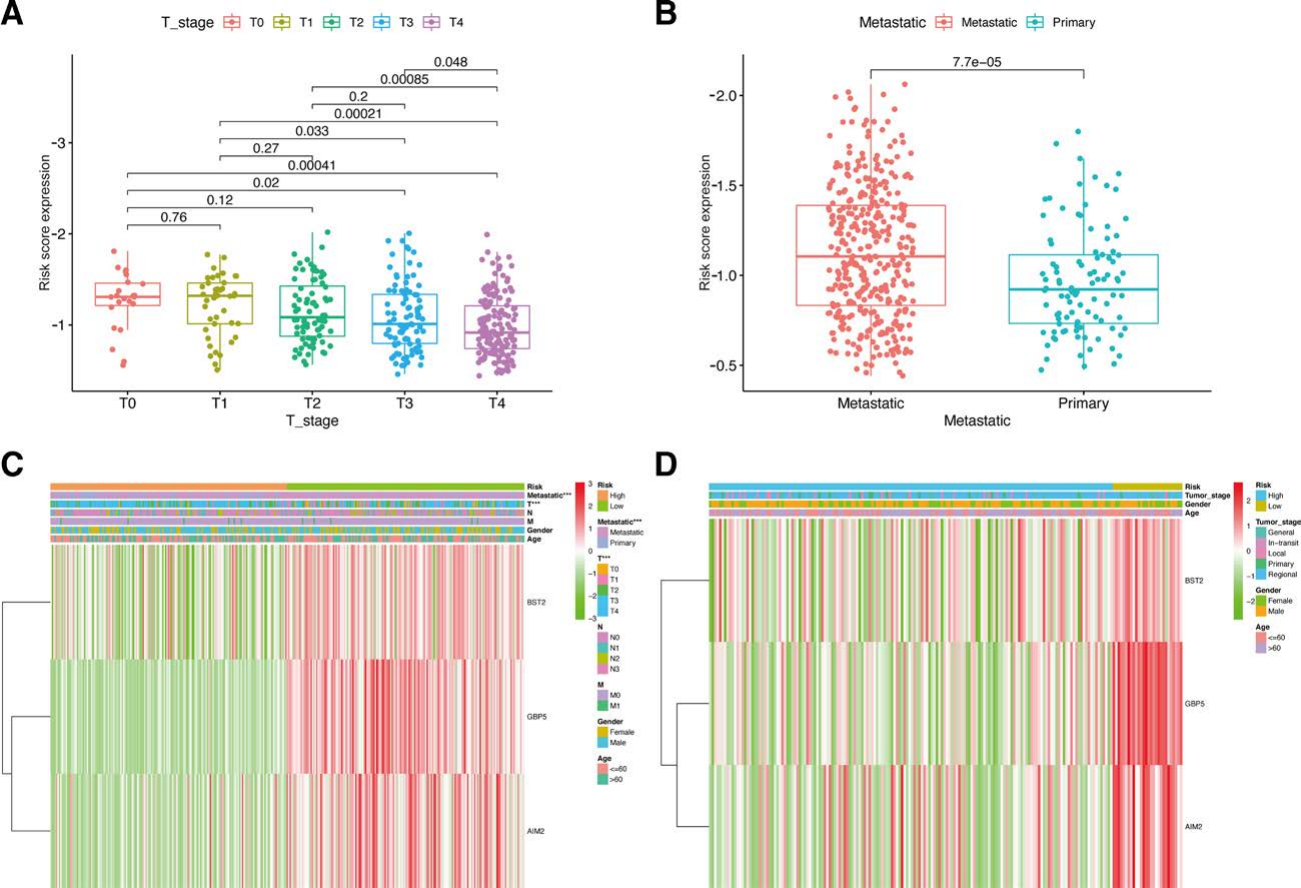
Association between risk score and clinicopathological parameters in patients with CM. Correlations between risk scores and T stage (A) and metastatic ability (B) in the TCGA cohort. Heatmap depicts the expression of pyroptosis genes and clinicopathological features according to risk subgroups using data from TCGA (C) and GSE65904 (D). TCGA = The Cancer Genome Atlas.

Interestingly, In the patients with CM, significant correlation between higher T stages and higher risk scores was observed (*P* < .05, Fig. 5A). Heatmaps of 2 CM cohorts were generated containing the gene expression of the 3 hub pyroptosis genes and the relationship between risk signature and tumor features (Fig. 5B and C). There were differences in subgroups regarding T stages in the TCGA-CM cohort (*P* < .05). These analyses suggest that our risk signature is connected to the development of CM.

### 3.5. Prognostic value of selected hub OS genes

**Figure 6.**
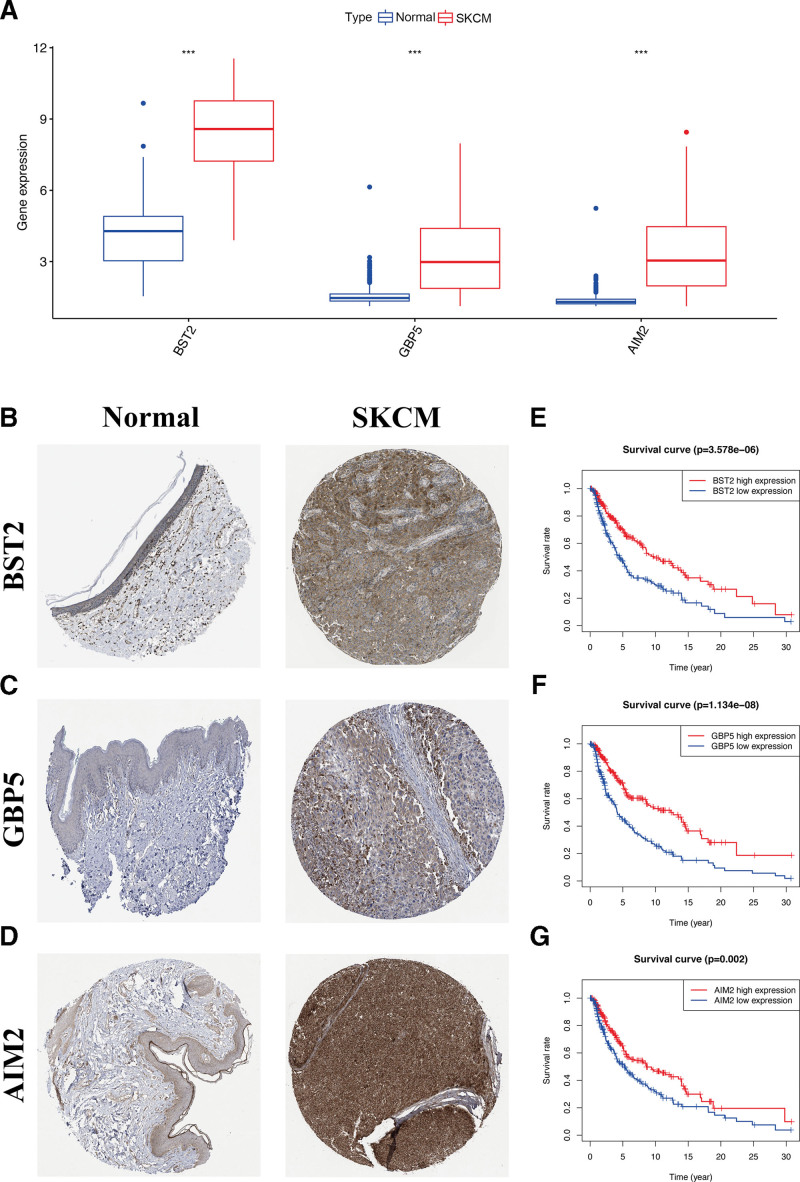
Prognostic value of selected hub pyroptosis genes. (A) The box plot shows pyroptosis genes using data from TCGA. The HPA database verified protein expressions of BST2 (B), CBP5 (C), and AIM2 (D) in CM. Kaplan–Meier analysis in TCGA cohort verified prognostic value of BST2 (E), CBP5 (F), and AIM2 (G) in CM. ***P < .001 relative to the normal group. CM, cutaneous melanoma, HPA = the human protein atlas, TCGA = The Cancer Genome Atlas.

Significantly elevated gene expression was found for pyroptosis genes *BST2, GBP5*, and *AIM2* in CM tissues when compared to that of normal tissues (Fig. 6A). These results were also confirmed by immunohistochemistry, which was obtained from the Human Protein Atlas (Fig. 6B–D). Next, the Kaplan-Meier survival analysis was used to examine the prognostic value of pyroptosis genes. This method showed that *BST2* expression was significantly involved in the CM patients’ prognosis (cutoff value = 8.572, *P* < .001, Chi-square = 22.172), *GBP5* (cutoff value = 2.991, *P* < .001, Chi-square = 32.597), and *AIM2* (cutoff value = 3.103, *P* = .002, Chi-square = 9.741) (Fig. 6E–G).

### 3.6. Functional enrichment analysis and nomogram construction

**Figure 7.**
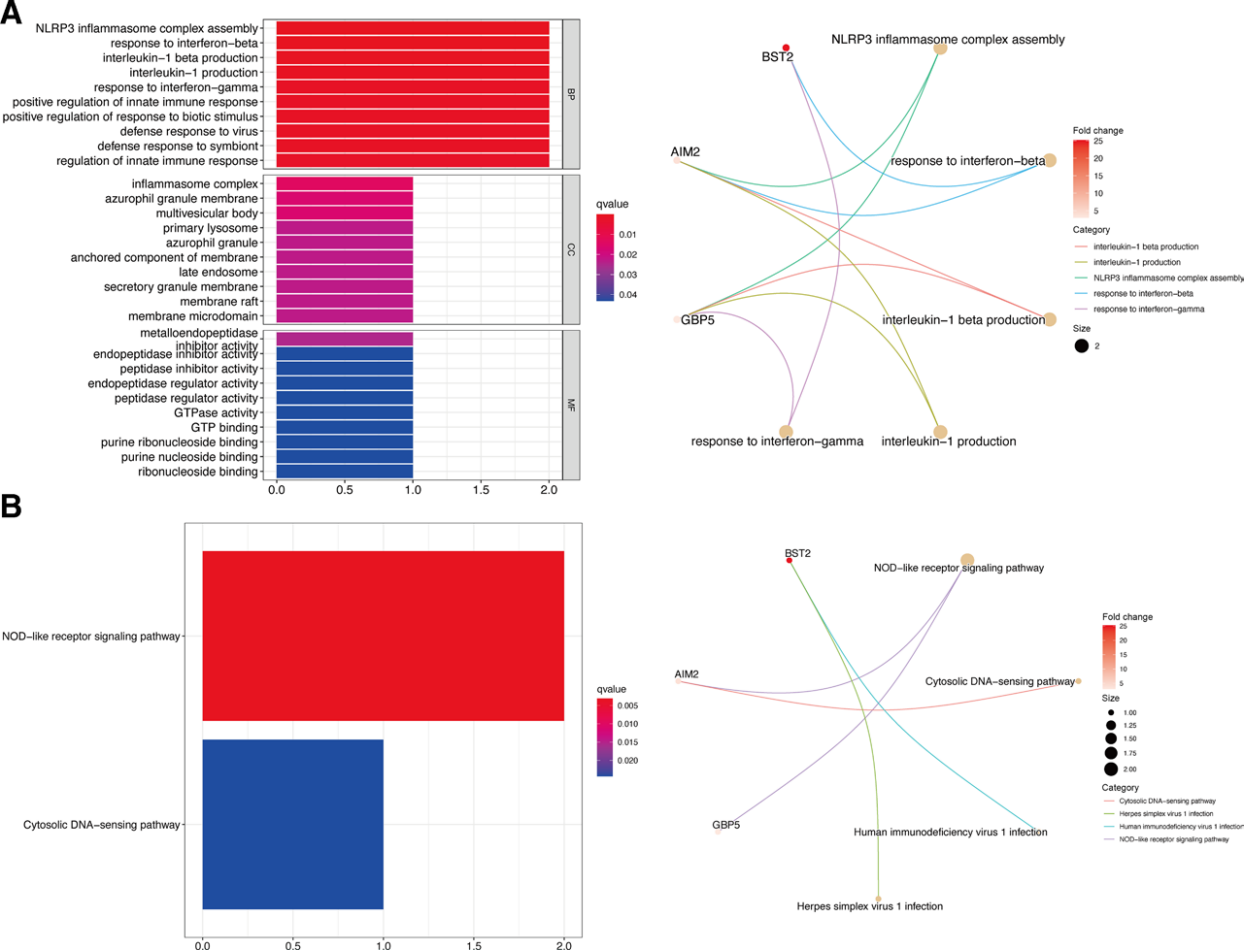
Functional enrichment analysis. (A) GO enrichment terms of hub pyroptosis genes in CC, BP, and MF. (B) KEGG enrichment terms of hub pyroptosis genes. The size of dot depicts the number of augmented genes. BP = biological process, CC = cellular component, KEGG = Kyoto Encyclopedia of Genes and Genomes, MF = molecular function.

Through the analysis of gene ontology enrichment, we observed significantly higher expression of the hub genes in NLRP3 inflammasome complex assembly, a response to interferon-β and -γ, and interleukin-1 production (Fig. 7A). Meanwhile, the Kyoto Encyclopedia of Genes and Genomes enrichment analysis indicated that pyroptosis genes were augmented in the NOD-like receptor signaling pathway and cytosolic DNA-sensing pathway (Fig. 7B). These results depict possible underlying mechanisms of pyroptosis genes in CM.

**Figure 8.**
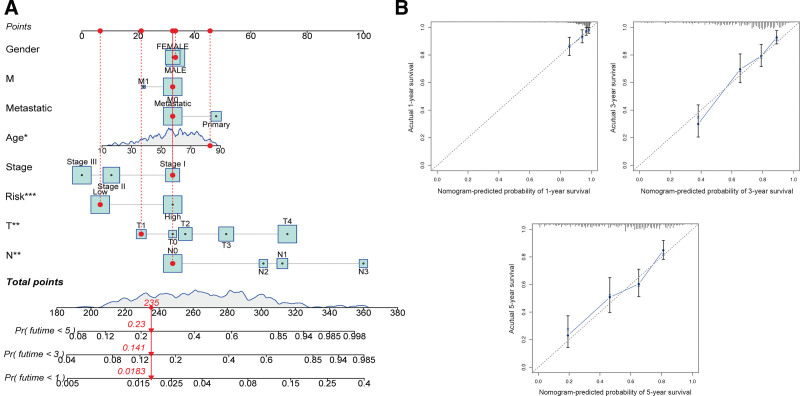
Development of a nomogram based risk scores and clinical characteristics. (A) Nomograms to predict the overall survival of CM patients at 1-, 3-, and 5-year using data from TCGA. (B) Calibration plot of the nomogram. TCGA = The Cancer Genome Atlas.

In addition, the risk signature was applied to construct a nomogram plot to predict patient outcome (Fig. 8A). Based on calibration plots, the constructed plot at 1, 3, and 5-year follow-up showed substantial agreement (Fig. 8B). Thus, the constructed nomogram may be used in the clinical management of CM patients.

## 4. Discussion

Despite the discovery of novel biomarkers in melanoma patients, more accurate markers are still needed for early detection and survival prediction. Here, we constructed a pyroptosis gene coexpression network that revealed that some pyroptosis genes were differentially expressed in CM patients. In addition, LASSO and univariate Cox regressions identified 3 prognosis-associated genes, namely *BST2, GBP5*, and *AIM2*. These hub genes were overexpressed in CM tissues. Moreover, they were found to be positively associated with the outcome of CM patients.

Abundantly expressed in several solid tumors, *BST2*, also known as CD317 and HM1.24, was shown to affect the invasion and progression of multiple myeloma.^[24]^ Studies reported that a *BST2*-blocking monoclonal antibody could lead to cellular cytotoxicity of multiple myeloma cells.^[25,26]^ Indeed, a *BST2*-associated humanized monoclonal antibody has been explored as immunotherapy to treat multiple myeloma.^[27]^ In parallel, a bioinformatics analysis discovered that *BST2* served as an important networking partner of the YWHAE gene,^[28]^ thus suggesting a role in melanocyte development and pigment formation. Another report has shown that *BST2* was significantly correlated with the clinical outcome of melanoma patients and lowly expressed in a high-risk subgroup,^[29]^ which is consistent with our study. However, the specific role of *BST2* in CM development and skin pigmentation is still unclear.

As the superfamily of INF-inducible guanosine triphosphate hydrolases, 7 guanylate-binding protein (GBP) genes were discovered.^[30,31]^ These genes can act as protective factors to control autoimmunity and infection and participate in the growth of various tumors via immune responses.^[32]^ For example, the CRISPR/Cas9 system knockout of GBP1 expression can effectively suppress the metastasis of prostate cancer.^[33]^ Indeed, GBP1 upregulation promotes brain metastasis of breast cancer via stimulating T lymphocytes.^[34]^ Moreover, GBP2 is related to a higher T-cell response and indicates a better prognosis in breast cancer patients.^[35]^ However, its promoter methylation can induce breast cancer progression.^[36]^ While considering the role of GBP5 in cell pyroptosis, a previous study confirmed that the expression of GBP5 is critical for the inflammasome; meanwhile, GBP5 could positively regulate the process of pyroptosis through the activation of caspase-1.^[37]^

In recent years, several studies have assessed the prognostic value of GBP genes in various cancers. GBP5 was associated with poor prognosis in breast cancer^[38]^ and served as a good prognostic marker in PD-L1 and PD-1 high-expressing basal-like breast tumors.^[39]^ A bioinformatics analysis reported that high expression of GBP1–5 was coincidentally and individually correlated with a favorable prognosis of CM patients,^[40]^ which indicates that GBP genes may be potential prognostic biomarkers.

As a member of PYHIN family, absent in melanoma 2 (AIM2) was not found in melanoma. AIM2 can induce cell pyroptosis to fight infections by binding to dsDNA and assembling the inflammasome.^[41]^ The sugar-phosphate backbone of dsDNA binds the positively charged HIN-200 domain through electrostatic interactions, which can relieve the self-oligomerization of the pyrin signaling domain and recruit adaptor protein PYD and CARD domain containing for the inflammasome activation.^[42]^ This process will activate caspase-1 and promote the release of potent proinflammatory cytokines IL-18 and IL-1β, that act in several diseases, including skin diseases,^[43]^ neuroinflammation,^[44]^ chronic kidney disease,^[45]^ and others. Concerning its effect in tumors, AIM2 was initially identified as a tumor suppressor gene in melanoma patients.^[46]^ This previous finding has been confirmed in our current study. Later explorations revealed that the antitumorigenesis effects of AIM2 are relevant to multiple types of cancer. For example, a study showed that AIM2 exerts an inflammasome-independent function in colorectal tumorigenesis.^[47]^ This gene can suppress the proliferation of colonic stem cells and induce cell apoptosis via the inhibition of PI3K/AKT and mTOR signaling pathways.^[48,49]^ Meanwhile, AIM2 can sense chemotherapeutic agents and ionizing radiation-induced DNA damage in bone marrow cells and intestinal epithelial cells and contribute to the therapeutic benefits of these therapies.^[50]^ Similar results are also reported for hepatocellular carcinoma, in which AIM2 displays anticancerous effects by inducing pyroptosis and suppressing mTOR pathways.^[51]^ AIM2 can also inhibit the migration and invasion of hepatocellular carcinoma cells by targeting the fibronectin-1 axis.^[52]^

However, studies also report a controversial protumorigenesis effect of AIM2 in selected types of cancer types, like cutaneous squamous carcinoma^[53]^ and nonsmall cell lung cancer.^[54]^ Indeed, AIM2 inflammasome activation can promote the proliferation of cutaneous squamous carcinoma cells by increasing the production of MMP-1 and MMP-13.^[53]^ In parallel, AIM2 leads to the pyroptosis of tumor-associated plasmacytoid dendritic cells with immunosuppressive effects in lung cancer,^[55]^ highlighting the significance of AIM2 in tumorigenesis. Even though BST2, GBP5, and AIM2 were already described to be linked with CM, no studies have assessed the prognostic value of these pyroptosis genes. In this study, we confirmed that the expression level of all these 3 pyroptosis genes were significantly negatively connected with CM patients’ overall survival. Our results provide novel data on the association of these pyroptosis genes and the prognosis of CM patients and identify biomarkers for personalized therapy.

Here, we provide a novel prognostic prediction signature that can be used as a prognostic marker, creating a pyroptosis-related risk signature for CM. Cox regression analyses revealed that this signature has a reliable prognostic value. Meanwhile, the risk signature successfully predicts the prognosis of CM patients, accompanied by increased accuracy compared to gender, age, T stage, N stage, M stage, TNM stage, and metastatic ability. Furthermore, through analyzing the clinical features and the risk signature’s relationship, we revealed the role of pyroptosis in CM carcinogenesis and progression. Indeed, correlations between our signature and T stage, which widely used when clinical evaluating, was observed in CM patients. Compared with T stage, the constructed risk signature showed a better prognosis value and could be used to predict CM growth.

Although 2 pyroptosis-associated gene signatures have been identified in CM,^[56,57]^ the selection of pyroptosis genes in these signatures was all based on a single bioinformatics analysis method, which lacks discriminatory ability for highly connected genes. Meanwhile, sole focus on the expression levels of individual genes ignores intergenomic epistasis. WGCN is used to evaluate the association between genes and phenotypic traits rather than focusing on individual gene expression.^[22]^ Furthermore, differential gene expression analysis of transcriptional data is another powerful tool providing changes in quantitative expression levels between 2 subgroups.^[58]^ In this study, candidate pyroptosis genes differentially expressed in CM tissues versus normal skin were identified using WGCNA and differential gene expression analysis to enhance the discriminatory ability of highly connected genes. Although there were still gaps with some signatures based on other genes, the results suggested that our analysis methods also had an effective prognostic performance in CM. In addition to transcriptome data that can be used for these analyses, we hypothesize that other omics data, such as proteome and metabolome, can be used similarly for tumor diagnosis and prognosis.

Nonetheless, despite these findings, this study has limitations. First, this is a retrospective analysis; thus, prospective approaches need to be performed to confirm our results. Second, more experimental data are needed to confirm our conclusions obtained from bioinformatics methods. Therefore, further mechanistic studies are needed to uncover the detailed mechanism linking the identified pyroptosis genes to the pathogenesis and progression of CM.

## 5. Conclusion

In conclusion, 3 hub pyroptosis-associated genes linked to outcomes of CM patients were identified by the construction of a coexpression network and several bioinformatics tools. We have successfully constructed a pyroptosis-associated prognostic signature with a potent predictive accuracy. This pyroptosis signature was used to predict the clinical outcomes of patients with CM. We believe that this study significantly contributes to improve the prognostic prediction of CM patients using a risk model based on pyroptosis genes.

## Author contributions

Conceptualization, data curation, formal analysis: Zhaoyang Shi; methodology: Jiaying Gu and Yi Yao; project administration, writing: Zhengyuan Wu.
